# Supplementary material for: Functional Tissue Units in the Human Reference Atlas
Source: bioRxiv. 2023 Oct 26:2023.10.16.562593. Preprint. [Version 3] doi: 10.1101/2023.10.16.562593 (PMC10614912; doi:10.1101/2023.10.16.562593)
Supplement: Supplement 1 [file NIHPP2023.10.16.562593v3-supplement-1.pdf]

bioRxiv preprint doi: https://doi.org/10.1101/2023.10.16.562593; this version posted October 26, 2023. The copyright holder for this preprint (which was not certified by peer review) is the author/funder, who has granted bioRxiv a license to display the preprint in perpetuity. It is made available under aCC-BY 4.0 International license.

Supplemental table

Table S1. Vascular pathways from the heart to each FTU and back to the heart
https://github.com/cns-iu/hra-vccf-ftu-supporting-information/tree/main/data
